# Dynamic Aspects of Macrophage Polarization during Atherosclerosis Progression and Regression

**DOI:** 10.3389/fimmu.2014.00579

**Published:** 2014-11-12

**Authors:** Michael Peled, Edward A. Fisher

**Affiliations:** ^1^The Marc and Ruti Bell Program in Vascular Biology, Department of Medicine, Leon H. Charney Division of Cardiology, New York University School of Medicine, New York, NY, USA

**Keywords:** macrophages, atherosclerosis, regression, cholesterol, polarization

## Abstract

It is well recognized that macrophages in many contexts *in vitro* and *in vivo* display a spectrum of inflammatory features and functional properties. A convenient system to group together different subsets of macrophages has been the M1 (inflammatory)/M2 (anti-inflammatory) classification. In addition to other sites of inflammation, it is now established that atherosclerotic plaques contain both M1 and M2 macrophages. We review results made possible by a number of recent mouse models of atherosclerotic regression that, taken with other literature, have shown the M1/M2 balance in plaques to be dynamic, with M1 predominating in disease progression and M2 in regression. The regulation of the macrophage phenotype in plaques and the functional consequences of the M1 and M2 states in atherosclerosis will also be discussed.

## Introduction

Atherosclerotic cardiovascular diseases, which include myocardial infraction and stroke, are the most common causes of morbidity and mortality in western society and will soon be the same world-wide. Atherosclerosis represents a failure to resolve the inflammatory response in the arterial wall initiated by the retention of apolipoprotein B (apoB)-containing lipoproteins (1). These lipoproteins are taken up by tissue macrophages, which ultimately become engorged with cholesterol (foam cells) and activated. The continuing stimulus of the entry and retention of apoB-lipoproteins fuels not only the accumulation of foam cells to form a plaque, but also the chronicity and amplification of the inflammatory response, which contribute to the vulnerability of some plaques to rupture and cause acute tissue ischemia. The central role of macrophages in atherosclerosis pathophysiology has, therefore, focused attention on their properties in plaque initiation and progression, and more recently, in regression (2–5).

The study of macrophages over the past decade is characterized by a remarkable expansion of knowledge concerning their origin, functional properties, and potential to both protect from and contribute to disease [e.g., see in Ref. (6, 7)]. Admitting the complexity of macrophage biology, for the purposes of this review, we have restricted ourselves to considering how aspects of macrophage polarization in the M1/M2 classification system (8) relate to atherosclerosis progression and regression. In this system, which is influenced by the Th1 and Th2 classification of lymphocytes, macrophages can be grossly divided into pro-inflammatory, M1 cells and anti-inflammatory, M2 cells based mainly on *in vitro* criteria (9). It is important to note, however, that while the classification of lymphocytes into Th1 and Th2 preceded the classification scheme of macrophages as M1/M2, the Th1 and Th2-like responses result from polarization of macrophages to M1 and M2 states, respectively. Furthermore, M1/M2 polarization is not dependent on T cells, as has been demonstrated in Rag KO and other immune deficient mice (8).

Polarization toward the M1 state is induced by several stimuli *in vitro*, including Toll-like receptor (TLR) ligands (such as lipopolysaccharide, LPS) and interferon γ (potential endogenous stimuli in atherosclerosis will be discussed below). M1 macrophages express several pro-inflammatory mediators, such as inducible nitric oxide synthase, tumor necrosis factor-a (TNF-a), interleukin-1b (IL-1b), IL-6, IL-12, and proteolytic enzymes. M1 macrophages have been found in both human and mouse atherosclerosis, and their secretion of the pro-inflammatory mediators is thought to maintain local inflammation and the degradation of extracellular matrix components, resulting in disease progression [e.g., Ref. (10)], and ultimately, unstable plaques in humans. As alluded to earlier, unstable plaques are at increased risk of rupture and causing thrombosis, resulting in myocardial infarction and stroke (11–14).

On the other side of the polarization spectrum, M2 macrophages are induced *in vitro* by Th2-type cytokines, such as IL-4 and IL-13. T regulatory cells (Tregs) have also been implicated in the induction of M2 polarization (15), possibly through IL-10. M2 polarized macrophages have been characterized by their expression of CD163, mannose receptor 1 (also known as CD206), FIZZ1, and high levels of arginase 1. In addition, they secrete anti-inflammatory cytokines, such as transforming growth factorβ, IL-1 receptor antagonist, IL-10, and have increased secretion of collagen. Based particularly on the role of M2 macrophages in wound healing, the combination of the factors they express are thought to be particularly suited for tissue repair, which is consistent with their increased presence in regressing plaques in mouse models of atherosclerosis (16, 17).

In addition to the M1 and M2 macrophages, oxidized phospholipids present in oxidized LDL induce a macrophage phenotype that is distinct from M1 or M2 phenotypes and that has been termed Mox; these macrophages are characterized by the increases in the expression of nuclear factor erythroid 2-related factor 2 (NRF2)-dependent genes and in reactive oxygen species, and are found in the progressing plaques (18). Their role in atherosclerosis regression, however, has not been established.

With this background, we will now turn to a more complete consideration of the inflammatory states of macrophages in atherosclerosis progression and regression.

## Atherosclerosis Progression

### Drivers of macrophage inflammation in progressing plaques

As mentioned above, M1 macrophages are thought to have significant roles in progressing and vulnerable plaques (19). As noted above, a potent inducer of the M1 state *in vitro* is LPS, which is part of the outer membrane of Gram-negative bacteria, and which binds and activates TLR4 (20). Dozens of studies have demonstrated associations among high-fat diet, the metabolic syndrome, and endotoxemia (i.e., increased levels of LPS in the plasma) (21–23). It appears that a high-fat diet, the consumption of which leads to metabolic syndrome in mouse models, also induces a change in the gut bacterial flora, which, in turn, causes an increase in the LPS plasma levels. Circumstantial considerations that support a link between the metabolic syndrome and LPS signaling include the increased cardiovascular risk in patients with the metabolic syndrome and the positive association of TLR4 activity with atherosclerosis progression in mice and human beings (24–28). Despite the evidence for association between LPS, atherosclerosis progression, and M1 polarization, a study in germ-free apoE-deficient mice on low-fat chow diet showed increased atherosclerosis progression (29), suggesting that some bacteria in the gut flora have an anti-inflammatory effect (30).

#### Cholesterol and macrophage activation

**Figure 1:**
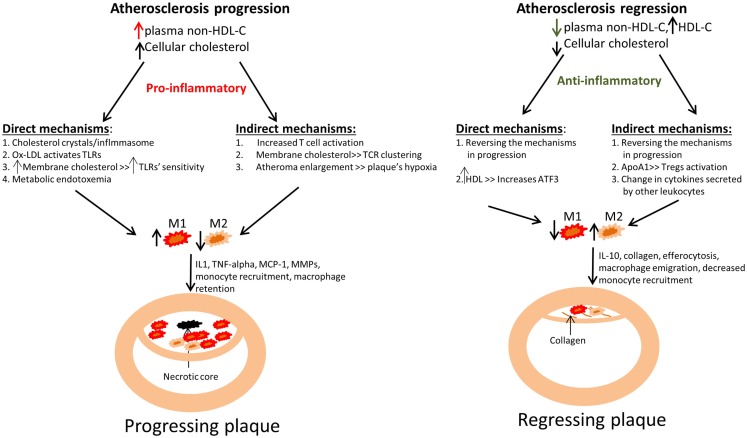
**Summary of how changes in plasma cholesterol level and in cellular cholesterol content affect macrophage polarization and kinetics in atherosclerotic plaque progression and regression**. Left panel: An increase in non-HDL cholesterol (mainly VLDL cholesterol and LDL cholesterol) in mouse models has been linked to an increase in monocyte recruitment into atherosclerotic plaques, with their subsequent polarization to M1 macrophages, which are retained. This ultimately leads to atherosclerotic plaque progression, as evident by plaque enlargement. The failure to clear dead macrophages by efferocytosis results in the appearance of the necrotic core. Right panel: An opposite effect has been demonstrated in atherosclerosis regression models, where a reduction in non-HDL-C or a selective increase in HDL-C (representing an increase in functional HDL particles) induces a decrease in plaque size and macrophage content (from decreased monocyte recruitment and macrophage retention), as well as enrichment in the expression of markers of the M2 state. Improved efferocytosis is also expected under these conditions, with shrinkage of the necrotic core. There is an increase in collagen content, likely from decreased MMP production by the macrophages. It is also likely that in a regression environment there are decreases in the secretion of inflammatory cytokines and chemokines by the macrophages as a result of the polarization of macrophages toward a M2-like state. The different mechanisms by which cholesterol can drive macrophage activation and polarization are divided into those direct – how cholesterol affects macrophages, and indirect – how cholesterol affects other cell types, for example by the secretion of pro-inflammatory cytokines from T cells.

The most accepted and robust risk factor for atherosclerosis is low-density lipoprotein-cholesterol (LDL-C). Thus, several studies have tried to understand how cholesterol can induce inflammation in general, and specifically to an activated state. The different mechanisms by which cholesterol can drive macrophage activation could be divided into those direct – how cholesterol affects macrophages, and indirect – how cholesterol affects other cell types through which activation could be induced, for example by the secretion of pro-inflammatory cytokines from T cells (Figure 1).

##### Direct mechanisms that link cholesterol to macrophage inflammation

Accumulation of cholesterol leads to the formation of crystals that are both intra- and extracellular. The presence of cholesterol crystals in early lesions in Apoe-/- mice was recently demonstrated (31, 32); in addition, it was shown that both macrophage engulfment of cholesterol crystals and *de novo* formation of intracellular cholesterol crystals activate the NLRP3 (NOD-, LRR- and pyrin domain-containing 3) inflammasome (33). Activation of NLRP3, in turn, results in the secretion of the pro-inflammatory cytokine IL-1β.This pathway appears to be necessary for atherosclerosis progression, as LDL receptor (Ldlr)-/- mice, transplanted with bone marrow cells deficient in IL-1β or in components of the NLRP3 inflammasome, had reduced plaque progression (31, 34).

A second direct mechanism that can explain macrophage activation by cholesterol is mediated through oxidized LDL. Oxidized LDL is present in both human and mouse atheromas. LDL oxidation is thought to be mediated by enzymes (such as 12/15-lipoxygenase and myeloperoxidase) and by free radicals that are abundant in the atherosclerotic plaque (35). Several studies have demonstrated that oxidized LDL can act as a ligand for both the scavenger receptors and TLRs on macrophages. Depending on the extent of oxidation, species of oxidized LDL were found to be agonists of CD14–TLR4–MD2 or CD36–TLR4–TLR6 complexes *in vitro*; these complexes can induce a pro-inflammatory signaling cascade involving IL-1 receptor-associated kinase 4 (IRAK4) (36, 37), myeloid differentiation primary-response protein 88 (MYD88) (28, 38) and other signaling proteins, culminating in activation of Nf-kB targets. Whole-body knock out mice of TLR2, TLR4 and some of the signaling proteins mentioned here were demonstrated to have reduced atherosclerosis progression (28, 39).

A third direct mechanism points toward the increase in plasma membrane cholesterol. The change in the microenvironment of TLRs (40, 41) renders them more sensitive to ligands, thereby heightening the inflammatory responses.

Despite the predominance of data that show a direct link between cholesterol and macrophage inflammation, there are a number of studies that show the opposite – an anti-inflammatory phenotype induced by intracellular cholesterol. The basis for this is rooted in the LXRs and PPARs, which are important nuclear receptors. The ligands for these receptors include lipids, and in addition to regulating many steps of their metabolism, LXRs and PPARs can also suppress inflammatory signaling in macrophages (42). For example, a recent study demonstrated that cholesterol-loading induced LXR-dependent downregulation of inflammatory gene expression in macrophages as a consequence of the accumulation of the cholesterol precursor desmosterol (43).

##### Indirect mechanisms that link cholesterol to macrophage inflammation

It has been long known that T cells participate in plaque inflammation (4). For example, using flow cytometry of whole aortic digests, Galkina et al. could demonstrate the presence of diverse cell-types of the adaptive immune system in the atherosclerotic plaque and the surrounding adventitia (44). Moreover, the same group showed that antigen presentation to CD4+ T cells in the arterial wall causes local T cell activation and the secretion of pro-inflammatory cytokines, which promote atherosclerosis by maintaining chronic inflammation and inducing foam cell formation (45). Other studies have shown a direct pro-inflammatory role of intracellular cholesterol in T cells, mediated by cholesterol-induced nano-clustering of T cell receptors (46). In addition, mice lacking T cells can have a significant reduction in atherosclerosis progression (47). Some studies, however, have demonstrated only a minor role for T cells in atherosclerosis progression (48).

Another indirect effect on macrophage polarization/activation by cholesterol is related to its being a major structural component of progressing plaques, so its ongoing accumulation will contribute to enlargement of the atheroma. This, in turn, is likely to contribute to a hypoxic environment because as the atheroma grows, the distance between the intimal cells from an oxygen supply will increase, particularly in mice, which have little capacity to form vasa vasorum. Hypoxia triggers a heavy reliance on glycolysis for energy production and it has been recently recognized that M1 cells are more glycolytic and M2 cells are more fatty acid oxidizing, and that factors that promote one pathway of energy generation over the other will promote the polarization state corresponding to the favored pathway (49). In addition, hypoxia can contribute to the formation of the necrotic core, a characteristic feature of advanced plaques that in humans increases the possibility of rupture (50). In response to the hypoxic conditions in the arterial wall, the development of vasa vasorum is enhanced, and this has also been related to atherosclerosis progression, possibly by the recruitment of monocytes to the plaque through this vascular route (51).

### Macrophage polarization and location in progressing plaques

Recent studies have found that although both M1 and M2 macrophage numbers are increased during human plaque progression, M1 macrophages were the predominant phenotype in rupture-prone shoulder regions, whereas M2 markers were predominant in the adventitia and in stable cell-rich areas the of plaque (19). In another study of human plaques, using the mannose receptor as a marker, M2 macrophages were found located far from the lipid core of the plaque (52).

In mouse plaques, M1 macrophages tend to be diffusely distributed in, and characteristic of, progressing plaques at the usual age that atherosclerotic mice are examined in detail (typically after 12–16 of the consumption of a high-fat, high-cholesterol diet, or ~16–20 weeks of age). Khallou-Laschet et al. have found in apoE-deficient mice of a similar age, but with less advanced atherosclerosis because they were maintained on a low-fat, chow diet that these early plaques were infiltrated by M2 macrophages, with M1 macrophages appearing later. Disease progression correlated with the dominance of M1 over the M2 phenotype (53). Based on serial histologic examination, they further propose that the M2 → M1 shift in balance was due to a phenotypic switch of the infiltrated cells, but the data to support this were indirect and did not exclude the possibility of replacement of macrophages by new ones with a different phenotype, or the local proliferation of M2-like tissue-resident cells, which has been reported in other contexts as well (54, 55). In regard to the location of the different macrophage phenotypes in the plaque, they show that M2 macrophages accumulated in the luminal side of the plaques in young mice, while at 55 weeks of age, both M1 and M2 labeling was evenly distributed across the plaque.

### Functional consequences of M1 polarization in progressing plaques

The secretion of a wide range of cytokines and chemokines (e.g., IL-1, TNFalpha, MCP-1) by M1 macrophages serves to further activate macrophages, as well as other cell types in the atheroma, such as endothelial and smooth muscle cells. There are also effects on cellular lipid metabolism. In one study of human plaques, macrophages with a marker of M2 macrophages had small lipid droplets and in studies *in vitro*, this was associated with a decreased ability to efflux cholesterol (52). In a study of murine macrophages, when the cells were polarized to the M1 state, there was downregulation of ABCA1 and reduced cholesterol efflux (56). If this were to happen *in vivo*, a vicious cycle would be formed – cholesterol accumulation would contribute to macrophage activation and M1 polarization, which would further induces cholesterol accumulation. Before concluding that this happens, given the divergent results on the effects of the M1 and M2 phenotypes on cholesterol efflux, more research in this area is clearly needed.

M1 macrophages also secrete chemokines (such as MCP-1) and cytokines (such as IL-12) that induce chemotaxis of other white blood cells (57, 58). In addition, M1 macrophages secrete several matrix metallo-proteinases (MMPs), such as MMP2 and MMP9, that can degrade the extracellular matrix in the plaque, which is thought to lead to destabilization and rupture. Indeed, MMPs were shown to co-localize with M1 macrophages in atherosclerotic plaques (59).

## Atherosclerosis Regression

Fatty streaks, the initial phase in the development of plaques, were found in children as young as 3 years of age (60), with well-established plaques developing by adolescence (61). Thus, while most studies are focused on the progression of atherosclerosis and finding means to delay it, the more frequent clinical scenario is that by the time the patient comes for treatment of cardiovascular risk factors, as a frequently middle-aged adult, he or she may already have a significant burden of atherosclerosis, making the optimal goal of therapy the induction of plaque regression.

Plaque regression can be defined in various ways, such as a reduction in plaque size, plaque cholesterol content, plaque macrophage number/percentage, or a decreased inflammatory state. Of course, multiple changes can occur simultaneously, but not in every case. For example, if the plaque macrophage content decreases, while collagen content increases, as we have observed experimentally in some models of regression [e.g., Ref. (16)], the size may not change, but there will be less inflammation and more stabilizing material. Nevertheless, though size changes may vary, one consistent finding in various mouse models of atherosclerosis regression in which the issue of macrophage polarization was examined, as will be summarized below, is that the plaque content of M1 markers decreased, while those of M2 markers increased [e.g., Ref. (16, 17, 62)].

### Atherosclerosis regression models and changes in plaque macrophages

Some of the currently available regression models include aortic arch transplantation model (17, 63), Reversa mouse model (16, 64), adenoviral gene transfer of the LDL receptor, apoA1 or apoE, a “hypomorphic apoE” model, administration of an inhibitor of MTP, and infusion of apoA-I (the major protein in HDL) or apoA-I mimetics (5, 65–69). By necessity, all models begin with a progression phase, in which the total plasma cholesterol, and in particular, LDL-C and VLDL-C, are very high. After a certain period of time, preferably at least 12–16 weeks of western diet (rich in saturated fat and cholesterol) in order to accelerate in apoE-deficient-based or enable in Ldl receptor-deficient-based models the development of a complex atherosclerotic plaque, regression is typically induced by a major change in the plasma lipid profile. This change is either a reduction of LDL/VLDL-C or an increase in apoA1/HDL, both of which would decrease the plaque content of cholesterol.

In the transplantation model, the plaque-containing aortic arch from a donor a*poE*-deficient (a*poE*^−/−^) or *Ldl* receptor-deficient (l*dlr*^−/−^) mouse fed a high-fat diet for 16 weeks is transferred into the abdominal aorta of a normo-lipidemic wild-type recipient mouse or an *apoE*^−/−^ mouse made transgenic in human *apoA1* (“*hA1/EKO*”) (17, 63, 70, 71). In either case, the regression of the plaque in the transplanted arch occurs within a few days. The advantage of the transplant model is that it can be used to test the effects of specific genes on regression by using knock out or transgenic strains, either for the donor or recipient mice, as well as to conveniently study leukocyte trafficking in and out of the plaques if the donors and recipients are mismatched in isoforms of the pan-leukocyte marker for CD45. The disadvantage lies in the technical difficulty in performing such a surgery in the mouse, thereby limiting the throughput. In addition, there is inherent inflammation induced by the surgery itself, which theoretically can affect the process of regression, though control transplants into a*poE*^−/−^ mice are used as a standard control for these and other effects related to the surgical procedures.

As noted above, HDL and its major protein, apoA1, can be increased by injections of apoA1, apoA1 mimetics, or an adenoviral vector expressing apoA1 (69, 72). In advanced atherosclerotic plaques of a*poE*^−/−^ mice, every other day injections of native human apoA-I over only 1 week led to atherosclerosis regression, as demonstrated by significant decreases in plaque lipid content, macrophage number, and an increase in collagen content; moreover, apoA1 injections led to a significant reduction in the plaques of inflammatory M1 and an increase in anti-inflammatory M2 macrophage markers, mannose receptor 1 and arginase 1 (73). Treating mice with a recombinant adenovirus encoding human apoA1 with relatively early atherosclerotic plaques resulted in a 70% reduction in aortic lesion area characterized by a significant decrease in the fraction of lesions occupied by macrophages and macrophage-derived foam cells. The inflammatory status of this population of cells was not reported (69).

Another example for plaque regression induced by an increase in HDL was shown with our collaborators using an inhibitor of microRNA-33 (miR-33). miR-33 suppresses HDL formation in the liver and its ability to efflux cholesterol from macrophages by suppressing the expression of cholesterol transporter ATP-binding cassette transporter 1 (*ABCA1*) (74). It was hypothesized that inhibiting it by an antagomir (anti-miR-33) would promote atherosclerosis regression. *Ldlr*^−/−^ mice with established plaques were treated with anti-miR-33 over 4 weeks. As expected, anti-miR-33 treatment led to increased reverse cholesterol transport through an increase in HDL levels and expression of *ABCA1* in the liver and macrophages. Consistent with that, and consistent with the apoA1 injection study, atherosclerotic lesions regressed by anti-miR-33 treatment, as shown by reduced plaque size, lipid and macrophage content, increased collagen content and a diminished inflammatory state of the macrophages in the plaque (75).

The Reversa (*Ldlr*^−/−^*ApoB100/100Mttpfl/flMx1Cre*^+/+^) mouse is a non-surgical regression model, based on the *Ldlr*^−/−^ mouse, in which the hyperlipidemia can be reversed by inducing the conditional knock out of the microsomal triglyceride transfer protein (*MTTP*) gene (64). MTTP is required for the proper assembly of VLDL, the precursor of LDL (76). The reversal of hyperlipidemia by inactivation of *MTTP* leads to regression of atherosclerosis over a few weeks accompanied by favorable changes in the composition of the atherosclerotic plaque. Again, plaque lipid content decreases, collagen content increases, and M1 markers are decreased while M2 markers are increased in the plaque macrophages (16). The advantage of the Reversa model is that it does not require any surgery in order to get extreme reduction in LDL/VLDL-C. In addition, it is important to note that unlike the transplant models, there is no increase in HDL after inducing the conditional knock out of the MTTP gene. This might be part of the reason for the reduced regression rate that can be seen in the Reversa model compared with the transplant model. The disadvantage of the model lies in the complicated genetic manipulations that were performed to create it – there are four different gene insertions/deletions in the Reversa mouse, and thus breeding it with another transgenic/knock out mouse to test the importance of a specific gene for regression is extremely time-intensive, making bone marrow transfer for myeloid-specific factors a more convenient manipulation.

The hepatic overexpression of *apoE* in *apoE*^−/−^ or *ldlr* in *ldlr*^−/−^ mice is two gene transfer strategies to induce regression, again by normalization of the lipid profile (5). The drawback of this method is a potential immune response of the host after the adenoviral gene transfer (77), which might complicate the interpretation of the inflammatory state of cells in the plaque. Also, especially with early versions of viral vectors, there can be limitations related to the duration or amplitude of expression.

Inducing regression just by a diet change, from western diet to chow, in the *ldlr*^−/−^ mouse model has also been tried. Many times, no significant changes have been observed, presumably because the plasma cholesterol levels remain elevated, and perhaps, the experiments were not continued long enough. In one recent report, we and our collaborators have observed a reduction in plaque macrophage content and inflammatory state over 4 weeks (78). Notably, these favorable changes were impaired by hyperglycemia, consistent with our previous report using Reversa mice (79).

#### Mechanisms for M2 macrophage enrichment in atherosclerosis regression

There are two major questions we will consider in this section – the origin of the M2 macrophages in regressing plaques and the mechanisms for their increase.

One possibility is that the re-balancing from enrichment in M1 to M2 markers in regressing plaques represents either a change in an individual cell, as can be accomplished *in vitro* by changing the cytokine environment, or as proposed to happen in the “early to advanced” plaque transition in *apoE*^−/−^ mice. Indeed, it is generally accepted that the phenotype of a macrophage is quite “plastic” and responsive to microenvironmental changes (9). It is also possible that M1-like macrophages leave and are replaced by M2-like cells, as occurs in wound healing. Support for this scenario is our demonstration, particularly in the aortic transplantation model, of emigration of macrophages from, and the ongoing recruitment of monocytes to, plaques in the regression environment (80). A third possibility is the induction of the proliferation of a resident population of yolk-sac-derived M2 macrophages, as observed in other settings (54). Support for this possibility is the recent demonstration in progressing plaques for macrophage proliferation (55).

It is tempting to speculate that the pro-activating direct and indirect effects of cholesterol described above are reversed under conditions of regression, under which the plaque content of cellular and extracellular cholesterol is typically reduced. Little experimental evidence, however, is available *in vivo* to prove this. Even for factors with known cholesterol-removing function, such as HDL, there could be lipid-independent reasons for their effects. For example, we have recently reported that murine bone marrow derived-macrophages (BMDM) not loaded with cholesterol will increase their M2 marker expression when incubated with HDL (81) [though in human monocytes this was not found to be the case (82)], and Latz and colleagues have found that HDL will induce in macrophages the transcriptional regulator ATF3, a repressor of a number of inflammatory factors (83). There is also a study in which injection of apoA1 reduced skin inflammation in *ldlr*^−/−^, *apoA1*^−/−^ DKO mice (which show signs of autoimmunity) by augmenting the effectiveness of the lymph node Treg cells (84); Tregs were shown to induce an M2 polarization in yet another study (15). In this example, there may have been a lipid-removal aspect in that *apoA1*^−/−^ mice are deficient in cholesterol efflux because of reduced numbers of HDL particles. Although unrelated directly to macrophage polarization, it has been shown that defects in cholesterol efflux can enrich the plasma membranes of monocyte precursors in the bone marrow with cholesterol, which results in their greater proliferation, circulating monocytosis, and increased entry into plaques of monocytes, which subsequently promotes atherogenesis (85). Importantly, we have recently found that this mechanism might also be related to the impairment of atherosclerosis regression in diabetes (7).

Turning to the issue of what regulates the enrichment in M2 macrophages in regressing plaques, there are no results to discuss at this time, but there are a number of possibilities. As mentioned earlier, potent cytokines that polarize macrophages are IL-4 and IL-13. These can be derived from a variety of leukocytes, namely Th2 lymphocytes, eosinophils, and basophils. Even if one or more of these types of leukocytes were the source of polarizing signals, there is still the mystery of how the change in the lipoprotein/lipid environment causes either the recruitment of the cells to the plaque or the stimulation of secretion from either pre-existing or newly recruited cells.

#### Functional contribution of M2 polarized cells to atherosclerosis regression

Another major and incompletely understood area is the requirement for, and the function of, the enrichment in M2 macrophages in atherosclerosis regression. Because their properties include tissue remodeling and inflammation resolution, it is tempting to attribute such changes in regressing plaques to M2 macrophages. This would be consistent with studies in which treatment of *ldlr*^−/−^ mice with IL-13 resulted in pre-existing plaques the following: an increase in collagen, a reduction in vascular cell adhesion molecule-1 (VCAM-1)-dependent monocyte recruitment, decreased macrophage content, and the induction of M2 macrophages, despite ongoing hyperlipidemia (86). Another reflection of the importance of the M2 macrophages for regression comes from our studies in diabetic Reversa mice, in which hyperglycemia impaired their enrichment in plaques despite lipid lowering and limited the favorable changes in macrophage content and inflammatory state (79).

The promotion of the resolution of inflammation by M2 macrophages in regressing plaques is likely a consequence of their secretion of IL-10. The plaque remodeling may represent at least two other properties of M2 macrophages, namely their secretion of collagen (87) and their enhanced capacity for efferocytosis (88). Efferocytosis is the clearance of apoptotic cells by macrophages. During atherosclerosis progression, the ability to store cholesterol in plaque macrophages in the form of cholesteryl ester lipid droplets wanes and free cholesterol accumulates, causing ER stress and apoptosis (89). If efferocytosis of the dying cells does not keep up with their formation, the cellular debris that are not cleared would be expected to accumulate and become a necrotic core. Indeed, in both mouse and human plaques, As plaques advance, the efferocytotic activity of their macrophages decreases (50). Thus, an enrichment in M2 macrophages with enhanced efferocytosis would be expected to clear apoptotic cells and thereby stop, and even reverse, necrotic core expansion.

## Concluding Remarks

During both the progression and the regression of atherosclerosis, macrophages have central roles. While macrophage phenotypes are diverse and form a continuum (90), we chose a simple and dichotomous approach in order to emphasize the differences between macrophage properties in atherosclerosis progression and regression. M1 macrophages predominate in progression and contribute to the inflammatory state, whereas M2 macrophages are enriched in many models of regression and appear to participate in inflammation resolution and plaque remodeling. Interestingly, M2 macrophages seem to have a beneficial role even when plasma cholesterol levels remain high [e.g., Ref. (17, 86)], and the opposite is also true – M1 macrophages, though activated by direct and indirect effects of cholesterol, have also been linked to an increase in atherosclerosis progression despite similar levels of plasma cholesterol, for example, in diabetes (91). Thus, there are complex interactions between macrophage phenotypes and plasma cholesterol levels, a situation likely to also exist with other known (and yet to be discovered) risk factors.

As noted throughout this review, there are many areas in which our knowledge of macrophage biology in plaques is inadequate. Yet, it is already clear that the inflammatory state of these cells is dynamically influenced by multiple metabolic, genetic, and pharmacologic factors. Deeper understanding of how these factors effect changes in plaque macrophages will likely advance the development of new strategies to reduce the huge burden of cardiovascular morbidity and mortality that persists with existing therapies.

## Conflict of Interest Statement

The authors declare that the research was conducted in the absence of any commercial or financial relationships that could be construed as a potential conflict of interest.
